# Reinforcement mesh repair for persistent pleural leakage following VATS lobectomy: a case report and clinical insights

**DOI:** 10.1186/s13019-026-04483-1

**Published:** 2026-06-21

**Authors:** Marco Lizwan, Ming Li Cynthia Chia

**Affiliations:** Department of Cardiothoracic Surgery, National Heart Centre Singapore, 5 Hospital Dr, Singapore, 169609 Singapore

**Keywords:** VATS lobectomy, Pleural leakage, Prolene^®^ mesh, Pleural effusion, Surgical reinforcement

## Abstract

Early chest drain removal and discharge are standard in enhanced-recovery protocols after video-assisted thoracoscopic surgery (VATS) lobectomy, but rare wound complications may arise. We report a 71-year-old man who underwent uneventful 3-port right VATS lower lobectomy. The utility access incision measured approximately 3.5 cm, was protected with a SurgiSleeve^®^ wound protector, and was closed in layers. No postoperative drain was placed through this site. The chest drain was removed on postoperative day 1 after absence of air leak and acceptable drainage output, and the patient was discharged on postoperative day 2. Two weeks later, serous pleural fluid leaked through the utility access wound. Conservative management with wound reinforcement, local compression, and empirical antibiotics failed. Re-exploration demonstrated clear pleural effusion without purulence, lung herniation, necrosis, or air leak. Selective reinforcement with polypropylene mesh anchored to the adjacent rib achieved wound stability, and the patient recovered without recurrence. Persistent pleural leakage through a VATS access wound is rare. In selected patients with persistent leakage after conservative management, and where infection has been reasonably excluded, mesh reinforcement may be considered as one feasible surgical option rather than a routine strategy.

**Supplementary Information:**

The online version contains supplementary material available at https://doi.org/10.1186/s13019-026-04483-1.

## Introduction

Video-assisted thoracoscopic surgery (VATS) has become the standard approach for anatomical lung resection, offering reduced postoperative pain, lower complication rates, and faster recovery compared with open thoracotomy [1]. The widespread implementation of ERAS protocols as also shifted has further accelerated postoperative recovery through early mobilisation, multimodal analgesia, and early chest drain removal in appropriately selected patients [2–4].

Delayed wound-related complications at VATS access sites are uncommon in contemporary practice, particularly because access incisions are small and layered closure is routinely performed. Lung herniation through an intercostal defect is a recognised but rare complication after thoracic surgery [5, 6]. By contrast, persistent pleural fluid leakage through a VATS access wound without parenchymal herniation is sparsely described.

We report a case of delayed pleural fluid leakage following VATS lobectomy that failed conservative management and was successfully treated with polypropylene mesh reinforcement. This case highlights diagnostic considerations, management strategies, and the role of chest wall reinforcement in selected patients.

## Case presentation

**Fig. 1:**
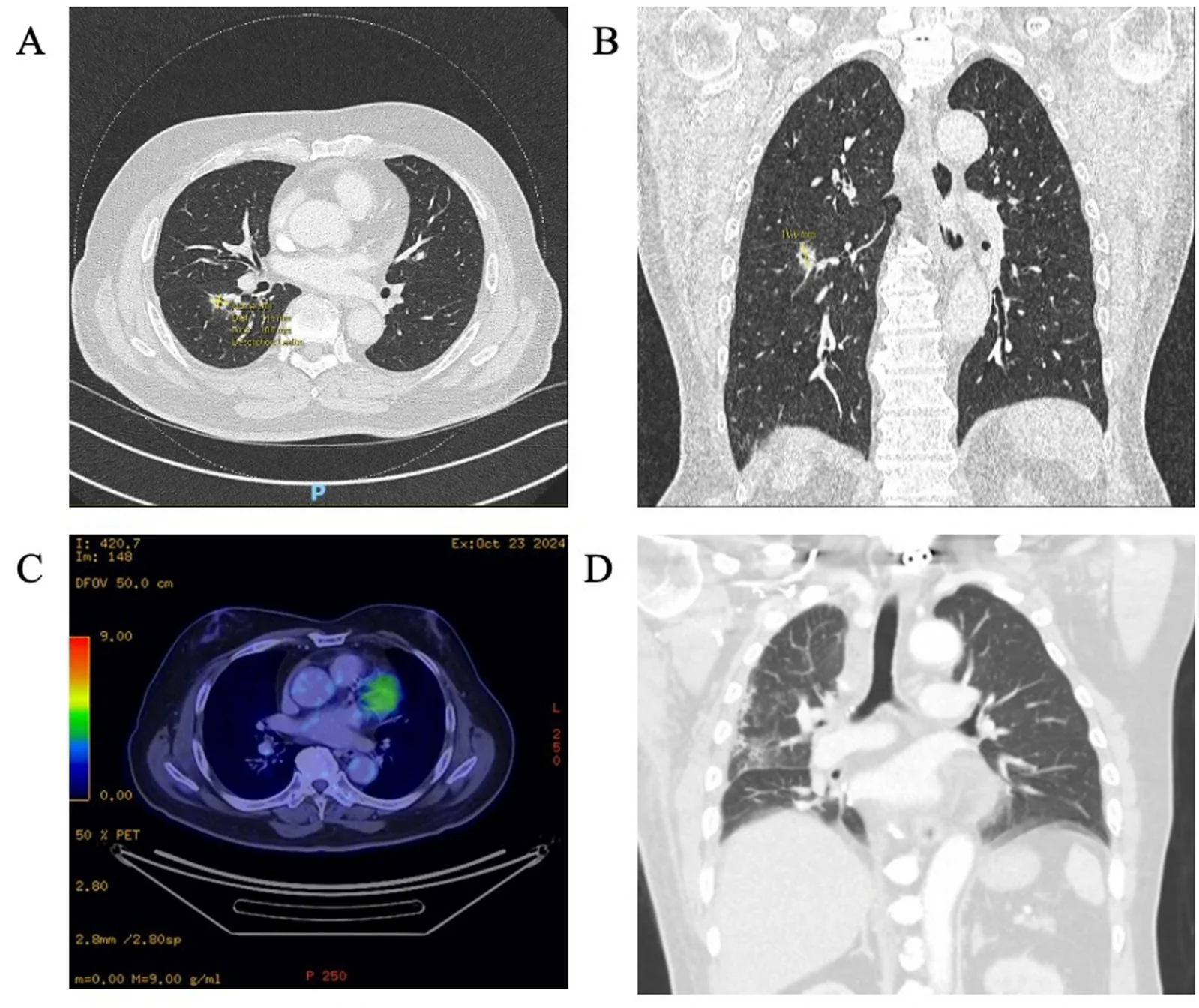
(**A**-**B**) CT thorax showed a spiculated sub-solid peri-bronchial nodule in the superior segment of the right lower lobe. (**C**) PET/CT scan showed a 2.2 cm right lower lobe nodule with mild FDG-avidity and no metastasis (**D**) CT thorax showed no definite rim-enhancing collection detected at the right chest wall with a new small right pleural effusion

A 71-year-old Eurasian male ex-smoker with comorbidities including hypertension, hyperlipidaemia, glaucoma, cervical spondylosis, previous spinal surgery, bilateral knee replacements and appendectomy, was referred for evaluation of a suspicious right lung nodule. Computed tomography (CT) of the thorax showed a right lower lobe nodule suspicious for malignancy (Fig. 1A-B). Positron Emission Tomography (PET)/CT scan revealed mild 18 F-fluoro-2-deoxy-d-glucose (FDG) avidity and no evidence of metastasis (Fig. 1C). Brain magnetic resonance imaging (MRI) was negative for metastasis.

The patient underwent standard 3-port right VATS lower lobectomy with mediastinal lymph node dissection [7]. The procedure was uneventful. Intraoperative air leak testing was negative. The VATS incision that subsequently developed postoperative leakage was the utility access port. A SurgiSleeve^®^ wound protector was used at this site, and the skin incision measured approximately 3.5 cm. At the completion of the initial operation, this access incision was closed in layers with two continuous layers of Vicryl^®^ sutures to the intercostal musculature and deep tissues, followed by subcuticular skin closure using Stratafix^®^. No postoperative chest drain was inserted through this access port; the drain was placed through a separate port site.

Postoperatively, the patient followed an institutional ERAS pathway, which included early mobilisation, multimodal opioid-sparing analgesia, and chest drain removal on postoperative day 1 after confirming absence of air leak and acceptable drainage output. Patient was discharged well on post-operative day 2.

**Fig. 2:**
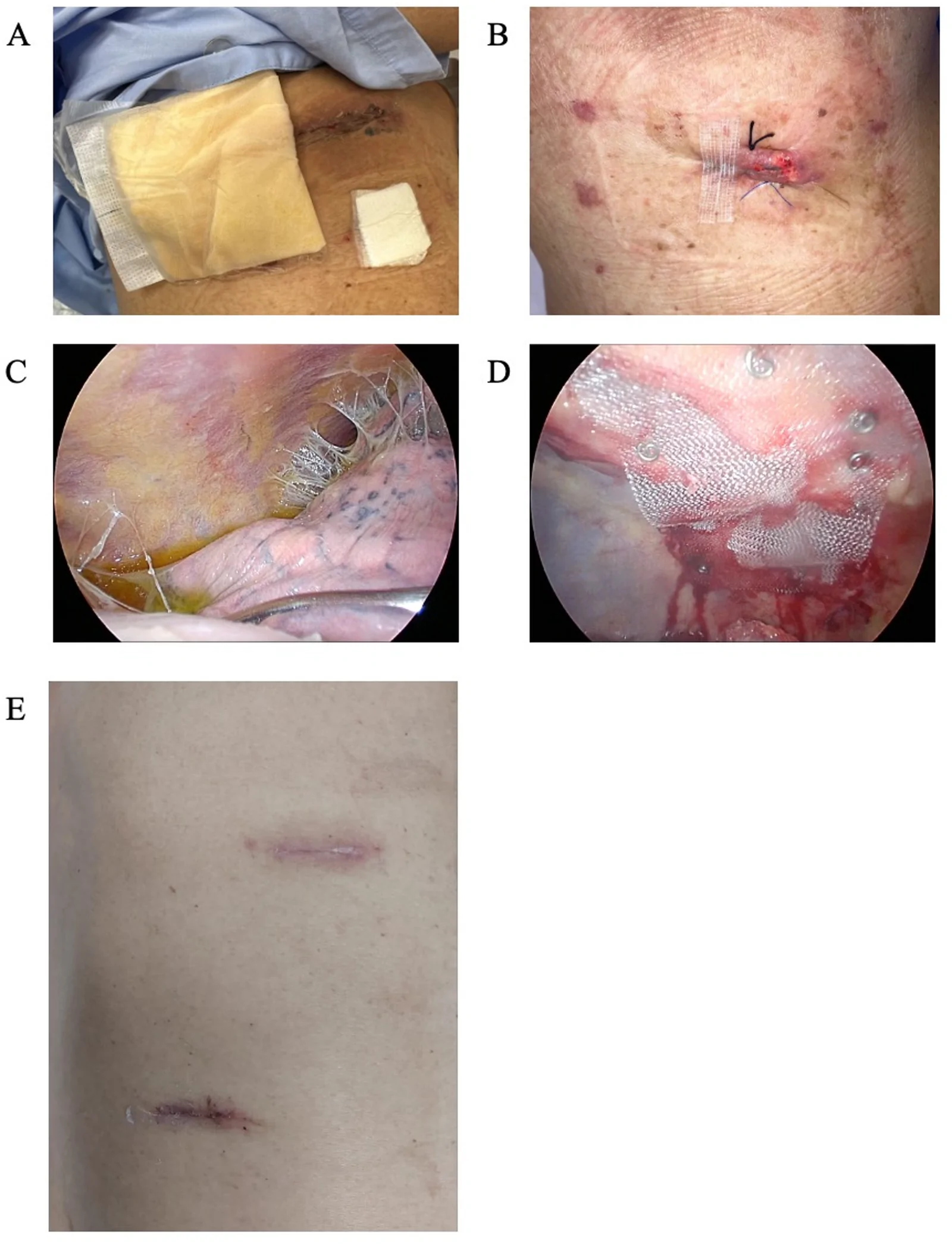
Clinical progression: (**A**) fluid leakage from previous VATS access wound, (**B**) temporary bedside wound reinforcement, (**C**) intraoperative findings of pleural adhesions and effusion without lung herniation, (**D**) and definitive repair with gentamicin-soaked Prolene^®^. (**E**) Follow-up revealed a well-healed wound with no further leakage

Two weeks later, the patient presented to the emergency department with visible serous fluid leaking from previous utility access wound (Fig. 2A). He was afebrile and haemodynamically stable. There was no local erythema, purulence, fluctuance, wound necrosis, palpable chest wall bulge, or clinical evidence of lung herniation. Laboratory investigations showed a mildly elevated C-reactive protein level without leucocytosis. CT thorax demonstrated focal soft tissue thickening at the surgical site and a new small right pleural effusion (Fig. 1D), without pneumothorax, rim-enhancing collection, gas locules, abscess, empyema, or extrathoracic lung herniation.

Given the absence of overt infection, initial conservative management was undertaken. This included bedside wound reinforcement with sutures (Fig. 2B), local compression, and a short course of empirical oral antibiotics. Despite these measures, persistent leakage continued over the following 24 h. The persistence of leakage after attempted closure raised concern for a deeper communication with the pleural space or local intercostal weakness rather than a superficial skin problem alone.

Given persistence despite conservative measures, the decision was made for VATS re-exploration. Intraoperatively, significant pleural adhesions were found, along with 300 mL of pleural effusion which was evacuated (Fig. 2C). No lung herniation or obvious air leak was observed. To prevent further leakage, Prolene^®^ mesh soaked in gentamicin was anchored to the rib (Fig. 2D, Video 1), mimicking a lung herniation repair technique [6]. Routine closure was then performed. This approach reinforced the thoracic wall defect, stabilised the wound, and provided local antimicrobial coverage to reduce the risk of postoperative infection. The surgical wound was closed routinely.

Patient was reviewed in the clinic 1-week and 2-week post-VATS exploration. During the clinic visit, his wound was healing well (Fig. 2E) and his repeat chest X-ray (CXR) was unremarkable.

## Discussion

This case illustrates a rare postoperative complication in which persistent pleural fluid leakage occurred through the VATS access incision after an otherwise uneventful lobectomy and early drain removal. Although VATS has significantly improved perioperative outcomes through smaller incisions, reduced pain, and shorter hospitalization, the widespread implementation of enhanced recovery after surgery (ERAS) protocols has also shifted postoperative practice toward early drain removal and same-week discharge [8, 9]. While this approach is safe and effective in the vast majority of patients, occasional delayed wound complications such as localized pleural leakage can arise. Awareness of such rare events is important to balance the benefits of accelerated recovery with patient safety.

In this case, the patient presented with delayed serous leakage two weeks after an initially uncomplicated recovery. Importantly, this presentation differed from surgical site infection, wound dehiscence, and lung herniation. Although CT demonstrated soft tissue thickening and pleural effusion, there were no imaging features of abscess or empyema. Clinically, the patient lacked systemic or local signs of infection, and intraoperative findings confirmed a clean surgical field.

The mechanism underlying pleural fluid leakage through a VATS access wound likely involves multifactorial processes. The proposed mechanism in this case remains a hypothesis rather than a directly demonstrated pathophysiological process. Following lung resection, minor pleural fluid accumulation is common, particularly if postoperative exudation exceeds the absorptive capacity of the pleura. In cases where the intercostal closure is slightly weakened – due to surgical dissection, local tissue oedema, or mechanical stress – fluid may track externally through the tract of least resistance. Increased intrathoracic pressure from coughing or movement can then perpetuate this leakage [10]. In this elderly patient with multiple comorbidities, delayed tissue remodelling and collagen cross-linking likely contributed to reduced wound tensile strength. The dynamic mechanical forces of respiration, particularly at lateral chest wall sites used for VATS access, may further stress the intercostal musculature and fascial closure, creating a small communicating tract. Although such micro-dehiscence may not be apparent intraoperatively, it can become clinically evident once pleural pressure differentials increase after discharge. However, no direct intraoperative test demonstrated a discrete pleurocutaneous tract, and therefore the mechanism should be interpreted cautiously.

It is essential to differentiate such cases from wound dehiscence, infection, or lung herniation. True lung herniation involves parenchymal protrusion beyond the thoracic wall, usually accompanied by palpable bulging or radiographic evidence of extra-thoracic lung tissue [11]. In contrast, pleural leakage produces fluid seepage without tissue extrusion and may be associated with small serous collections on imaging rather than herniated parenchyma. Initial management should remain conservative, focusing on wound re-suturing, local compression, and empirical antibiotics if infection is suspected. Most small leaks resolve spontaneously as granulation tissue develops. However, persistent leakage despite conservative measures suggests a structural defect in the intercostal space or failure of the fascial seal, warranting surgical re-exploration.

Initial conservative management remains appropriate, particularly when infection is uncertain. In this patient, bedside suturing, compression, and antibiotics were attempted. However, continued leakage indicated failure of conservative therapy and suggested an underlying structural defect requiring definitive intervention.

In this case, VATS re-exploration confirmed pleural adhesions and effusion but no herniation or air leak. Given the persistence of leakage despite bedside repair, reinforcement was required to ensure closure stability. Prolene^®^ (polypropylene) mesh was selected to reinforce the access wound due to its established durability, tensile strength, and long-term safety profile in chest wall reconstruction [12–14]. Anchoring the mesh to the adjacent rib effectively redistributed mechanical tension across a wider surface, preventing recurrence and promoting fibrous tissue incorporation for long-term stability. While mesh implantation introduces a foreign body, its use in a clean operative field with careful patient selection carries a low infection risk. The decision to proceed was supported by the absence of clinical, radiological, and intraoperative evidence of infection. Gentamicin soaking was used only as an adjunctive measure and not relied upon as primary infection prophylaxis, consistent with evidence that topical antibiotics alone are insufficient to prevent mesh-related infection [15]. The main purpose of the mesh in this case was mechanical reinforcement in a clean field, not antimicrobial therapy. The choice of polypropylene mesh was selective and should not be considered routine for all VATS wound leaks.

Alternative strategies, such as muscle flap advancement or multilayer fascial closure, may suffice in cases with limited leakage or robust local tissue. However, in an elderly patient with fragile musculature and prior wound stress, mesh reinforcement provides an added safety margin without significant additional morbidity.

Clinically, this case underscores the need for vigilance following early discharge under ERAS protocols, as patients may develop delayed wound-related complications. Judicious chest drain management remains crucial; while early removal reduces discomfort and hospital stay, drain duration should be individualized, especially in older patients or those with borderline output. Whether early chest drain removal contributed to this complication cannot be definitely determined. Early drain removal is a cornerstone of ERAS pathways and has been shown to be safe in appropriately selected patients [2, 4]. However, it is plausible that residual pleural fluid may exert pressure on a marginally healed access wound in selected individuals. Rather than challenging ERAS principles, this case underscores the importance of individualised postoperative decision-making and vigilance for rare delayed complications. The case should therefore not be interpreted as evidence against ERAS or early drain removal, but rather as a reminder that rare delayed wound events may still occur despite appropriate postoperative management.

Persistent pleural leakage is rare and likely underreported, as mild cases may resolve spontaneously or remain unrecognized. This report contributes to the limited literature on thoracic wall complications unrelated to air leaks or infection. Further studies could evaluate the incidence of such leaks in ERAS populations and compare the outcomes of mesh versus non-mesh approaches. Additionally, the potential role of bioabsorbable or biologic meshes warrants investigation, as these materials may offer equivalent support with improved tissue integration and reduced long-term stiffness.

## Conclusion

Persistent pleural fluid leakage through a VATS access wound is rare and should be suspected when delayed serous drainage persists despite initial wound reinforcement, particularly when imaging demonstrates pleural effusion without abscess or lung herniation. Conservative management remains the initial approach in stable patients. Mesh reinforcement should not be routine, but may be a feasible option in carefully selected cases when leakage persists, infection has been reasonably excluded, and local structural weakness is suspected.

## Supplementary Information

Below is the link to the electronic supplementary material.

Supplementary Material 1: Video 1. Video-assisted thoracoscopic surgery (VATS) showing Prolene® mesh anchoring for unusual complication of persistent pleural leakage post-VATS lobectomy.

## Data Availability

No datasets were generated or analysed during the current study.
